# Dental Caries Prevalence among 12–15 Year Old Palestinian Children

**DOI:** 10.1155/2014/785404

**Published:** 2014-10-01

**Authors:** Maen Mahfouz, Albina Abu Esaid

**Affiliations:** Department of Orthodontics and Pediatric Dentistry, Dental College, Arab American University, P.O. Box 240, Jenin, Palestine

## Abstract

*Objective.* To measure the distribution of dental caries in a group of Palestinian adolescents. *Material and Methods.* A sample of 677 individuals of both sexes (411 were females and 266 were males) their ages ranged from 12 to 15 year old randomly selected from schools in northern west bank in Palestine. Clinical examination was performed on all the subjects focusing on the index DMFT, representing the number of teeth that were either decayed, missing or with extraction indicated, or restored. *Results.* The prevalence of dental caries in the permanent dentition was 54.35% and was the highest in 15 age 75.75% in comparison to the other ages (12, 13, and 14) (40.57%, 41.76%, and 60.47%), respectively. The mean DMFT for the sample was 5.39 ± 2.85525 while the mean DMFT for different age groups (12–15) was 5.52 ± 2.766, 5.58 ± 2.745, 5.23 ± 3.304, and 5.23 ± 2.606, respectively. The prevalence of dental caries was higher in females with DMFT 5.39 ± 2.854 than males with DMFT 5.26 ± 2.891. *Conclusion.* High prevalent dental caries was found among Palestinian adolescents and higher in females than males. Strict preventive programs should be implemented. Further research with large samples required to include all adolescents from Palestine.

## 1. Introduction

Dental caries is one of the most prevalent diseases afflicting human beings and persists till date as a challenge to the medical and dental profession in particular and the society in general. Information on epidemiological figures of dental caries is a fundamental requirement which updates our knowledge on changing trends of the disease, its treatment needs and helps in understanding ways and means to prevent its onset, limit its progression, and consequences [1].

Oral health is now recognized as equally important in relation to general health. The major oral health problems around the world are generally considered to be dental caries and periodontal diseases. Previous studies show that most individuals seek dental care with complaints of pain mainly because of tooth ache related to dental caries [2].

Age of 12 years has been universally accepted as global monitoring age for caries since all permanent teeth except third molars would most likely have erupted by this age. By the age of 15, the dietary habits of the individuals are more or less established and the permanent teeth have been exposed to the oral environment for 3–9 years, thus making the assessment of caries prevalence even more meaningful at this age [3]. In addition this particular age group of people from 12 to 15 year old has the longer outdoors stay at this age leading to greater consumption of inbetween meals snacks, cariogenic diet, and consequently to be considered at high risk in terms of dental caries.

Measuring dental caries prevalence among Palestinian adolescents is extremely important to establish a baseline data which is essential for oral health planners to apply intervention programs in schools.

The aim of this paper is to measure the distribution of dental caries among 12–15 year old adolescents of northern part of Palestine.

## 2. Materials & Methodology

This study was carried out on 677 individuals of both sexes (411 were females and 266 were males); their ages ranged from 12 to 15 years. This sample was randomly selected from different schools in four main governorates in northern west bank in which individuals had no previous history of receiving any specific organized preventive treatment. The schools visited in four main governorates (Jenin, Tobass, Qbatia, and Alzababdeh) were chosen according to the list of Palestinian Ministry of Education. The sample involved in this study was divided into 4 groups depending on age to be expressive about dental caries status in different age groups in adolescence.

Age of 12 years has been universally accepted as global monitoring age for caries since all permanent teeth except third molars would most likely have erupted by this age. By the age of 15, the dietary habits of the individuals are more or less established and the permanent teeth have been exposed to the oral environment for 3–9 years, thus making the assessment of caries prevalence even more meaningful at this age [3].

Adolescents were examined in their respective schools seated in ordinary chair under adequate daylight and facing away from direct sunlight with the help of plain mirrors, standardized dental probes. World Health Organization (WHO) index was used to measure the prevalence of caries activity (2013) [4].

Statistical assessment was carried out using SPSS program version 20 to do descriptive statistics like mean, standard deviation and proportion was used to describe caries prevalence.

## 3. Results

This study aimed to measure the distribution of dental caries among a group of Palestinian adolescents from 12 to 15 year old in northern part of Palestine. The sample size was 677 involving different age groups.

Table 1 shows the percentage and number of source population whereby the samples (677) were drawn. Jenin Governorate represents the highest percentage which is 45% in comparison to other governorates Tobass, Qbatia, and Alzababdeh which are 25%, 20%, and 10%, respectively.

Table 2 shows the distribution of the sample according to the source of population, age, and sex. According to the source of population the sample was randomly selected from different schools in four main governorates. Accordingly, the sample is divided into four age groups from 12 to 15 year old according to the age and the sample also was divided into groups according to the sex in which each age group has both sexes; the female group size was greater than male group size in different age groups.

Table 3 shows the prevalence of dental caries in the permanent dentition which was 54.35%, as well as the prevalence of dental caries in 15 age which was the highest 75.75% in comparison to the other ages (12, 13, and 14) (40.57%, 41.76%, and 60.47%), respectively. Table 3 shows the mean DMFT for the sample which was 5.39 (SD 2.85525) while the mean DMFT for different age groups (12–15) was 5.52 (SD 2.766), 5.58 (SD 2.745), 5.23 (SD 3.304), and 5.23 (SD 2.606), respectively.

Table 4 shows the prevalence and severity of dental caries in different age groups (12–15 year old) for females. The highest percentage of dental caries prevalence for females was found in age 15 in comparison to other age groups while the lowest percentage for females is found in age 13. The highest DMFT score in females was in the age group 13 in comparison to other age groups while the lowest DMFT score was in the age group 15.

Table 5 shows the prevalence and severity of dental caries in different age groups (12–15 year old) for males. The highest percentage of dental caries prevalence for males was found in age 15 in comparison to other age groups while the lowest percentage for males was found in age 12. The highest DMFT score was in the age group 12 in comparison to other age groups while the lowest DMFT score was in the age group 13 (Table 5).

The prevalence of dental caries was higher in females with DMFT 5.39 (SD 2.854) than males with DMFT 5.26 (SD 2.891). The total DMFT score for all age groups was 5.39 (SD 2.85525) while the total of the caries prevalence of all age groups was 54.35%.

## 4. Discussion

The study reported here represents the first comprehensive analysis that includes a group of adolescents from 12 to 15 year old in northern part of Palestine. Oral health is an integral part of general health in spite of the fact that it is the most neglected one. The lack of awareness, limitation of access to the dental specialist, and underestimation of the preventive measures, even among the educated class of the society, have placed Palestinian adolescents among the most disease prone nations as shown by Tables 1, 2, 3, 4, and 5.

The results showed that the prevalence of dental caries in the permanent dentition was 54.35% (Table 3), and the prevalence of dental caries was the highest in 15 age 75.75% in comparison to the other ages (12, 13, and 14) (40.57%, 41.76%, and 60.47%), respectively. The mean DMFT was higher comparing with the global indicator of caries monitoring which was 5.39 (SD 2.85525) while the mean DMFT for different age groups (12, 13, 14, and 15) was 5.52 (SD 2.766), 5.58 (SD 2.745), 5.23 (SD 3.304), and 5.23 (SD 2.606), respectively (Table 3).

In this study the dental caries prevalence in comparison to other studies was lesser like study done by Shingare et al. who found that caries prevalence in Indian school children in age group 11–14 was 73% while in this study caries prevalence was different and ranged from 40.57% to 60.47% for age groups 12–14 [5] and a study done by Ahmed et al. and Rajab et al. on school children from Bagdad and Amman, respectively, found that dental caries prevalence in 12 year old age group was 62%, 45.5% which is different and higher than this study which found dental caries prevalence at age 12 group is 40.57% [6–8]. This finding is in contrast with a study done by Moses et al. who found the dental caries prevalence is 44.62% among 12–15 age group which is lesser than this study [9–11].

In the current study the prevalence of dental caries was higher in females 62.28% than males 42.10%. This finding corresponds with the study carried out by Okeigbemena who mentioned that the prevalence of dental caries in females was higher than males [7] and another study conducted by Shingare et al. found that the prevalence of dental caries in females was higher than males [5]. In contrast Moses et al. found that there was an increase in caries prevalence in boys compared to females [9].

## 5. Conclusion

1. High prevalent dental caries was found among Palestinian adolescents from age 12 to 15 years especially in females. The reasons for this would mainly be lack of dental awareness, motivation, ignorance, poor oral hygiene, improper tooth brushing techniques, and inadequate exposure to fluorides. Other contributing factors could be improper dietary habits, longer outdoors stay of children at this age leading to greater consumption of inbetween meals snacks, cariogenic diet, and nutritional deficiencies.
2. Different preventive measures should be applied to different ages and genders based on the findings that their caries prevalence and severity were different.
3. Strict preventive programs should be implemented and awareness of the adolescents' personal health measures is a must.
4. Further research and investigation with large samples required to include all adolescents from middle and southern Palestine as well as a wide group of Palestinian population.

## Figures and Tables

**Table 1:** Shows the percentage and number of source population whereby the samples (677) were drawn.

Governorate	Percentage	Number
Jenin	45%	305
Tobass	20%	135
Qabatia	25%	169
Alzababdeh	10%	68
Total	**100%**	**677**

**Table 2:** Shows the distribution of the sample according to the source of population, age, and sex.

Age group	Governorate	Female	Male	Total
12	Jenin	35	23	175
	Tobass	24	19	
	Qabatia	29	18	
	Alzababdeh	13	14	

13	Jenin	24	21	170
	Tobass	26	16	
	Qabatia	28	19	
	Alzababdeh	24	12	

14	Jenin	29	21	167
	Tobass	23	14	
	Qabatia	28	19	
	Alzababdeh	23	10	

15	Jenin	42	18	165
	Tobass	21	9	
	Qabatia	31	15	
	Alzababdeh	11	18	

		411	266	677

**Table 3:** Shows the prevalence and severity of dental caries mean DMFT in different age groups (12–15 year old).

Age group	Total number	Affected number	Caries prevalence	DMFT (Mean ± SD)	Number of teeth remaining
12	175	71	40.57%	5.52 ± 2.766	3997
13	170	71	41.76%	5.58 ± 2.745	3929
14	167	101	60.47%	5.23 ± 3.304	3655
15	165	125	75.75%	5.23 ± 2.606	3132
	677	368	54.35%	5.39 ± 2.85525	14713

**Table 4:** Shows the prevalence and severity of dental caries and mean DMFT in different age groups (12–15 year old) for females.

Age group	Total number	Affected number	Caries prevalence	DMFT (Mean ± SD)	Number of teeth remaining
12	101	50	49.50495%	5.28 ± 2.167	2443
13	102	44	43.1372%	6.05 ± 2.803	2426
14	103	70	67.96116%	5.31 ± 3.500	2276
15	105	92	87.6190%	5.21 ± 2.654	2186
	411	256	62.28%	5.39 ± 2.854	9331

**Table 5:** Shows the prevalence and severity of dental caries and mean DMFT in different age groups (12–15 year old) for males.

Age group	Total number	Affected number	Caries prevalence	DMFT (Mean ± SD)	Number of teeth remaining
12	74	21	28.37837%	6.10 ± 3.846	1554
13	68	27	39.70588%	4.81 ± 2.512	1501
14	64	31	48.4375%	5.03 ± 2.858	1378
15	60	33	55%	5.30 ± 2.506	946
	266	112	42.10%	5.26 ± 2.891	5379
